# Eosinophilic esophagitis in the “atopic march”: dupilumab as an “umbrella” strategy for multiple coexisting atopic diseases

**DOI:** 10.3389/fmed.2024.1513417

**Published:** 2025-01-21

**Authors:** Nicola Lutzu, Agnese Favale, Mauro Demurtas, Stefano Del Giacco, Sara Onali, Massimo Claudio Fantini

**Affiliations:** ^1^Department of Medical Science and Public Health, University of Cagliari, Cagliari, Italy; ^2^Azienda Ospedaliero-Universitaria di Cagliari, Cagliari, Italy

**Keywords:** esophagitis, atopic march, anti-IL4, anti-IL13, eosinophils, dupilumab, eosinophilic esophagitis

## Abstract

Dupilumab is a monoclonal antibody targeting interleukin-4 and interleukin-13, approved for the treatment of multiple T2 diseases and more recently for Eosinophilic Esophagitis (EoE). EoE is a chronic T2 inflammatory disease, believed to be a member of the “atopic march”, due to multiple similarities with other atopic diseases, ranging from epidemiology to genetics and pathophysiology. Although often co-existing in the same patient, these diseases are still treated as separated entities by different specialists, resulting in polypharmacy and chronic use of steroids. Thus, a shared-decision approach by a multidisciplinary team composed of different specialists might improve clinical management and outcomes. Yet, prospective data on the effectiveness of dupilumab as a single agent for multiple T2 inflammatory diseases are lacking, since only few case reports and small studies have been published so far reporting outcomes in patients affected by multiple T2 diseases. The purpose of this review is to illustrate the rationale and clinical evidence supporting the possibility of using dupilumab as a single therapeutic agent in those patients affected by multiple T2 diseases in addition to EoE.

## 1 Introduction

Eosinophilic esophagitis (EoE) is a chronic, antigen-driven, T2 disease, characterized by eosinophilic infiltration of the esophagus, impaired epithelial barrier function and tissue remodeling. This chronic inflammatory process can ultimately progress to fibrosis and esophageal dysmotility, (1) which manifest clinically with dysphagia and food impaction. Recently, EoE has been recognized as part of the “atopic march” (defined as “the natural history of allergic manifestations as they develop during infancy and childhood”), (2) together with other T2 diseases historically considered as a group: Bronchial Asthma (BA), Allergic Rhinitis (AR), Atopic Dermatitis (AD), and food allergies (FA) (3). Until recently, all these diseases lacked efficacious second-line alternatives after the failure of multiple conventional therapies, often causing chronic use of systemic and topical steroid (4, 5). For this reason, new biologic drugs targeting shared T2 pathogenetic mechanisms have been developed, such as dupilumab, an anti IL4Rα monoclonal antibody filed for different T2 conditions including EoE. Dupilumab offers the yet unexplored possibility to manage multiple diseases with one single therapy, thus potentially reducing healthcare-related costs and improving both patients’ adherence to therapy as well as health-related quality of life (hrQoL). However, despite EoE patients carry a 2.8 to 5.1-fold higher risk than the general population to develop concomitant T2 diseases, (6) these conditions are still managed independently, exposing one single patient to polypharmacy prescribed by different specialists. Moreover, EoE often remains unrecognized and underdiagnosed, especially when the clinical presentation resembles gastroesophageal reflux disease (GERD) (7). Accordingly, the ability of immunologists and dermatologists to recognize “red flags” for EoE and investigate for its presence could be decisive since, while the advanced therapeutic choice for EoE is still limited to dupilumab, more options are already available for BA, AD and chronic rhinosinusitis with nasal polyps (CRSwNP) (8). In this context, the presence of concomitant EoE might skew the therapeutic choice favoring dupilumab over other biologic agents, thus avoiding polypharmacy and its related drawbacks.

Thus, the aim of this review is to sensitize multiple specialists dealing with the “atopic march” on the importance of screening for EoE whenever red flags are present, hopefully promoting a multidisciplinary approach with the intent to co-manage different T2 diseases through an “umbrella” strategy with a single biologic agent.

## 2 Eosinophilic esophagitis

### 2.1 Epidemiology of EoE

EoE is an emerging disease as clearly showed by a rapid increase in incidence and prevalence in the last decades, partially because of a growing awareness of the disease but also for a true increase of its incidence (9, 10). According to a recent meta-analysis by Hahn et al. (11) the estimated overall prevalence of the disease is 40.04 cases per 100,000 inhabitants, while the global pooled incidence is 5.31/100,000/year. EoE is more common in adults than children (incidence rate 4.95/100,000 persons-year vs 7.20/100,000 persons-year in children vs adults, respectively), while the pooled prevalence by gender is higher in males than females (111.09/100,000 persons-year vs 32.83/100,000 persons-year, respectively) (11). Male predominance could be explained by the presence of a single nucleotide polymorphisms in the Thymic Stromal Lymphopoietin (TSLP) gene and its receptor, localized on Xp22.3 and Yp11.3, which has been detected only in male EoE patients (11).

### 2.2 Epidemiology of EoE related to other atopic diseases

EoE has frequently been considered as an atopy-associated disorder. In a multicenter single visit registry involving 705 patients with documented diagnosis of EoE, 91% of patients reported concomitant allergic disorders (12). A meta-analysis by González-Cervera et al. (6) outlined that AR, BA and eczema were significantly more common among patients with EoE compared with control subjects. Of note, 1 year after EoE diagnosis, 63.5% patients reported to suffer from at least another concomitant atopic disease, whom prevalence at 12 months reached approximately 44.7, 27.1, 25.2, and 16.9%, for BA, AR, AD, and FA, respectively (8). The association between EoE and atopy is further supported by retrospective studies showing seasonal variation in the number of new EoE diagnosis and symptom flares (13–16). Accordingly, a recent cross-sectional retrospective cohort study found lower response rates to food elimination diet (FED) during pollen seasons (17). Together, these findings support a possible role of aeroallergens in the pathophysiology of EoE, with swallowing of inhaled allergens as a possible additional explanation.

### 2.3 EoE: clinical presentation

Clinical manifestation of EoE is highly heterogeneous and mainly depends on age at presentation (18). In the pediatric age, the clinical presentation is often insidious, being characterized by non-specific signs and symptoms like failure to thrive, food refusal, abdominal pain, nausea, vomiting and reflux-like symptoms, while dysphagia and food impaction are less common (14, 19–22). In this age group, this faded symptomatology often prevents prompt investigation and delays EoE diagnosis. Symptoms related to esophageal dysmotility, rigidity and reduced distensibility, such as dysphagia, food impaction, chest pain and heartburn, occur more often in adults than children (14, 19, 23–26). Among these, dysphagia is the most common symptom, occurring in up to 70% of adult EoE patients (24). Ricker et al. (25) reported that among 100 patients with non-obstructive dysphagia, 22% had an unrecognized and undiagnosed EoE. Therefore, EoE should always be suspected in patients presenting with dysphagia. EoE is also the most frequent cause of food impaction in young adults and children, considering that almost 50% of patients who undergo esophagogastroduodenoscopy (EGD) for food impaction have an undiagnosed EoE (19, 23, 27, 28). Nevertheless, EoE-related symptoms are not specific to EoE, and other conditions like GERD or esophageal motility disorders may have the same clinical presentation (7). Diagnosis may be challenging when EoE presents with reflux-like symptoms resembling GERD, considering that both conditions may respond clinically to an initial trial of Proton Pump Inhibitors (PPI), postponing the need for endoscopy. Moreover, EoE patients often adopt compensatory eating behaviors described with the acronym “IMPACT”: Imbibe fluids with meals, Modify food (cutting into small pieces, pureeing), Prolong meal times, Avoid hard texture foods, Chew excessively, Turn away tablets/pills (29, 30). This may further increase the time interval before gastroenterologist referral, contributing to diagnostic delay, that ranges from 36 months up to 7 years (7, 12, 31, 32). Accordingly, all these symptoms can be considered as “red flags” of EoE, and they should be properly questioned and further investigated, especially when presenting in patient with other concomitant T2 diseases in whom the probability of EoE diagnosis is high. On the other hand, the GI specialist should be able to recognize key symptoms (i.e., wheezing, chronic cough, mouth breathing, nasal congestion, sneezing, eczema, itch) suggestive of extraesophageal atopic diseases. Nevertheless, to date, a list of “red flags” has not been defined and validated, though a clear checklist of symptoms could favor the multidisciplinary care, which, could reduce diagnostic delay by earlier referral to the best suited specialist.

### 2.4 EoE: diagnosis

The current diagnostic criteria are defined by European guidelines and the AGREE consensus (33, 34) and include symptoms of esophageal dysfunction, peak eosinophil count ≥15 eosinophils per high power field (eos/hpf) on properly collected esophageal biopsy specimens, and the exclusion of other local or systemic causes of esophageal eosinophilia. The updated diagnostic criteria are shown in Table 1. Additional histologic features that reinforce the diagnosis include eosinophil micro abscesses, basal zone hyperplasia, dilated intercellular spaces (spongiosis), eosinophil surface layering, papillary elongation, and lamina propria fibrosis, which are included in two EoE-specific validated scores, the EoE Histologic Scoring System (EoEHSS) and the EoE Histologic Remission Score (EoEHRS) (35, 36). Collins et al. (35) showed that, compared with peak eosinophil count alone, EoEHSS can better discriminate treated from untreated patients, while EoEHRS correlated more strongly with symptoms (i.e., dysphagia) (36). These findings suggest that EoEHSS and EoEHRS might be more accurate than peak eosinophil count alone to assess disease activity and treatment response. Nevertheless, the use of these scores in clinical practice is still limited due to their complexity.

**TABLE 1:** Updated diagnostic criteria by the AGREE consensus (33).

EoE diagnostic criteria	Supportive findings
Clinical symptoms suggestive for EoE	Concomitant atopic conditions should increase suspicion for EoE Endoscopic findings of rings, furrows, exudates, edema, stricture, narrowing, and crepe paper mucosa should increase suspicion for EoE
Esophageal eosinophilia ≥15 eos/hpf on esophageal biopsy	Eosinophilic infiltration must be limited to the esophageal mucosa
Exclusion of other systemic or local conditions that may cause or contribute to esophageal eosinophilia	.

EoE, eosinophilic esophagitis; eos/hpf, eosinophils per high power field.

Endoscopy is pivotal for the diagnosis of EoE due to the requirement of biopsy sampling and histologic evaluation. Moreover, typical endoscopic features included in the validated EoE Endoscopic Reference Score (EREFS; Edema, Rings, Exudates, Furrows and Strictures) (37) may be observed in EoE patients. These features are not necessary for the diagnosis but may support clinical suspicion. In fact, up to 10–32% of EoE patients may present a normal mucosal appearance at endoscopy, thus, especially when the clinical suspicion is high, biopsy specimens should be properly collected even in the presence of normal mucosa (38–42). Currently, collection of a minimum of 6 biopsy samples from mid-proximal and distal esophagus is recommended (41–44). In EoE patients, Nielsen et al. (45) estimated a probability to detect ≥15 eos/hpf of 63, 98, 99, and >99%, for 1, 4, 5, and 6 biopsy samples, respectively. However, in adult patients presenting with refractory reflux-like symptoms but lacking dysphagia or typical EoE features, routine biopsies are not recommended, given the very low diagnostic power of histology in this context and the low prevalence of EoE in this group of patients (43, 46–48). On the other hand, in PPI-responsive EoE, the diagnostic yield of histology performed during treatment is very low, considering that PPIs can induce histological remission in almost 50% of EoE patients (49). Thus the criteria of ≥15 eos/hpf may not be met. Accordingly, in patients with ongoing PPIs therapy in which the clinical suspicion for EoE is high, the British guidelines and the more recent Italian consensus suggest PPIs withdrawal for at least 3 weeks prior to EGD and biopsy sampling (43, 44).

### 2.5 EoE: conventional treatment

The therapeutic approach of EoE still represents a challenge. While the short-term therapeutic goals include the histologic, endoscopic and symptomatic improvement, in the long-term goals should be focused on maintenance of remission and avoidance of disease progression and disease-related complications. Indeed, current evidence show that EoE is a progressive disease if left untreated (50). The progression and evolution into a fibro-stenotic phenotype is associated with the persistence of dysphagia and a higher risk of disease-related complications including food impaction (1, 7, 9, 32, 50–52). The overall time of untreated disease has been found to be directly proportional to the risk of strictures formation and food impaction (50). Indeed, the risk for stricture formation increases by 9% for each year of untreated EoE and diagnostic delay was found to be the major predictive factor, (32) highlighting the importance of prompt diagnosis and early treatment.

Although clinical and histological relapses may occur during maintenance even after effective induction therapy, higher rates of relapse have been reported after treatment discontinuation (53–56). Consistently, Dellon et al. (55) found that almost two thirds of patients in remission had symptom recurrence within 1 year after therapy discontinuation while Greuter et al. (56) observed that symptom relapse occurred in up to 80% of patients, with a median time of 22 weeks after treatment cessation, thus highlighting the importance of maintenance therapy in EoE patients.

First-line treatment include dietary interventions and medical therapy. Current guidelines suggest against using allergy test to guide dietary elimination, considering that dietary restrictions based on food sensitization profiles are less effective in lowering eosinophilic count with respect to other dietary interventions, including elemental diet and FED (43, 57, 58). With regards to medical therapy, the current British and American guidelines as well as the more recent Italian consensus, recommend PPIs and swallowed topical corticosteroids (STCs) as first-line treatment (43, 58, 59).

Despite the demonstrated efficacy of STCs, PPIs and dietary intervention in EoE management, in a multicenter real-world European cohort including 589 patients affected by EoE, these treatments induced clinical and histologic remission or response in only 80.7, 69.2, and 41.7% of patients respectively (60). These data demonstrates that 20–60% of patients are refractory to conventional therapies and, similarly to other atopic disease, justify the need for the development of more advanced therapeutic strategies.

## 3 Shared genetic and immunological mechanisms in the “atopic march”

The “atopic march” model has been historically used to describe the natural history of T2 diseases. This model outlines the possible trajectories of subsequent allergic diseases development, starting from AD, which is often the first manifestation of the atopic march early in life, followed by BA, AR and/or FA, in a variable and bidirectional way (61, 62). The link between EoE and other atopic diseases has been hypothesized almost 20 years ago, when EoE has been defined as the “asthma of the esophagus” (63, 64) but only recently EoE has been proposed as a late component of the “atopic march” (62, 65). Several reasons support this hypothesis. First, EoE shares similarities in the epidemiology with other T2 diseases, including family history of atopic disorders, the co-occurrence of multiple atopic diseases in the same patient (6), and the seasonal variability of symptoms in aero-allergen sensitized patients (9, 13). Secondly, there is evidence of common genetic predisposition shared by EoE and other atopic diseases (66–72). Finally, the pathophysiological process characterized by a polarized immune response into the T2 pathway, with increased production of related cytokines, partly overlaps between EoE and the other diseases considered part of the “atopic march” (5, 67, 70).

The pathogenesis of EoE, as well as of the other T2 diseases, is the result of a complex interplay of genetic, immunological, and environmental factors.

### 3.1 Shared genetic predisposition

The role of genetic susceptibility in the development of T2 diseases has been confirmed by the association of several genes with an increased risk to develop atopic diseases including EoE (66, 71, 73–76). Moreover, in the last decades, gene-expression studies led to the identification of an EoE-specific transcriptomic signature, ultimately leading to the definition of the EoE Diagnostic Panel (EDP), a panel including 94 differently expressed genes that accurately discriminate EoE from non-EoE patients (77–79). Most of the genes included in EDP belong to the epidermal differentiation complex (EDC), which includes genes involved in the maintenance of the homeostasis and integrity of the epithelial barrier, frequently linked to an increased risk for other atopic conditions in which epithelial barrier dysfunction plays a pivotal role (61, 66, 71, 73–76, 80, 81). Of note, several genes are similarly dysregulated in AD, BA and EoE, namely filaggrin, desmoglein-1, claudin 1, cathepsin C, histamine receptor H1, plasminogen activator urokinase receptor, and suppressor of cytokine signaling 3 (70, 82–84). Finally, single nucleotide polymorphisms of TSLP gene have been equally correlated with increased odds of EoE, BA, AD and CRSwNP (66, 72, 82, 85–87).

### 3.2 Shared immunologic pathways

**FIGURE 1:**
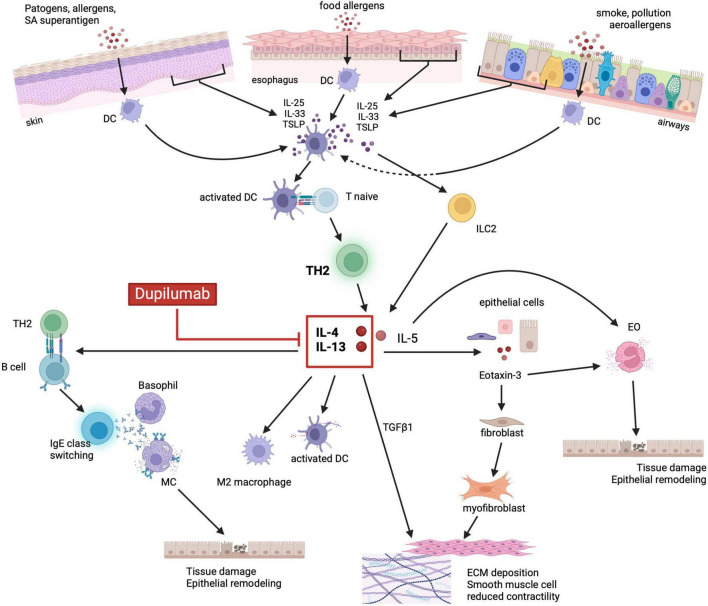
Shared immunopathologic mechanisms of the atopic march. Environmental factors (i.e., food, aeroallergens, infections) and the related tissue damage can stimulate the epithelium to release alarmins such as IL-33, IL-25 and TSLP. Alarmins, in turn, activate both ILC2 and DCs which drive naïve T cells toward a Th2 differentiation and production of IL-5, IL-4 and IL-13. IL-5 promotes eosinophils activation and degranulation, whereas IL-4 and IL-13 stimulate the release of eotaxin-3 by epithelial cells. Eotaxin-3 together with IL-5, ultimately lead to eosinophils recruitment, activation, and degranulation. IL-4/IL-13 also promote DC activation, M2 macrophage polarization, IgE class switching by activated B cells and consequently MC and basophils degranulation. Cytotoxic proteins released by granulocytes lead to tissue damage, barrier dysfunction and epithelial remodeling. Finally, IL-4/IL-13 stimulated release of TGFβ1, in synergy with eotaxin-3, promote fibroblast activation and differentiation into myofibroblast, ultimately leading to ECM deposition and smooth muscle cells reduced contractility. Dupilumab, by binding to IL-4Rα, can inhibit the activation of the JAK/STAT6 signaling pathway by IL-4 and IL-13, thus blocking the aforementioned downstream inflammatory processes. EO, eosinophils; DC, dendritic cells; M2, M2 macrophages; MC, mast cells; ILC2, innate lymphoid cell type 2; IL, interleukin; TLSP, thymic stromal lymphopoietin precursor; TH2, T helper 2; TGFβ1, transforming growth factor β1; ECM, extracellular matrix. Created with BioRender.com.

In addition to genetic predisposition, the diseases belonging to the “atopic march” share overlapping molecular pathways which constitute what is defined as “the T2 inflammatory response”. The activation of the same innate and adaptive immune cells sub-types that release similar cytokines, has been described in the different organs targeted by atopic diseases (70, 88). In genetically predisposed individuals, the exposure to some environmental factors (i.e., food and aeroallergens) triggers the activation of the T2 inflammatory response, through epithelial sensitization leading to tissue injury. This mechanism similarly occurs in the esophageal mucosa of EoE patients, in the skin barrier of AD patients and in the bronchial mucosa of BA patients (67, 88–91). In response to the mucosal damage, epithelial cells release different cytokines, including IL-25, IL-33 and TSLP, known as “alarmins”, which promote the activation and proliferation of innate lymphoid cells type 2 (ILC2) and drive the differentiation of naïve CD4 T cells into Th2 cells (85, 88, 92–100) (Figure 1). The activation of ILC2, Th2 and dendritic cells (DC) by the alarmins stimulates the expression of IL-4, IL-13, IL-5, IL-9, IL-18, IL-15, tumor necrosis factor α (TNF-α), transforming growth factor β1 (TGF-β1) and the TNF-related cytokine LIGHT (5, 67, 92, 101–106). All these cytokines are largely expressed in EoE as well as in the other diseases of the “atopic march”. Among these cytokines, IL-4 and IL-13 are considered to have a key role in orchestrating the T2 immune response, (101–103, 107, 108) although in EoE several studies have demonstrated that IL-13 is expressed in higher concentrations compared to IL-4 (103, 109).

Both IL-4 and IL-13 share the IL-4Rα, whose binding causes the activation of the STAT6 signaling pathway, that upregulates the expression of the *CCL26* gene with the subsequent increased production of eotaxin-3 and eosinophils recruitment (110–113). The IL-13-STAT6-induced release of eotaxin-3, in synergy with TGF-β1, activates quiescent fibroblasts, stimulates their differentiation into myofibroblasts, and reduces the esophageal and bronchial smooth muscle contraction causing dysmotility of the esophagus (114, 115). Moreover, besides the production of eotaxin-3, the activation of the IL-13-STAT6 signaling pathway increases DC activity, promotes maturation of M2 macrophages, drives B cells into IgE class switching, and, through the downregulation of EDC expression, leads to the disruption of the epithelial barrier integrity leading to tissue remodeling (5, 81, 92, 116–118). Additionally, IL-13-STAT6 activation induces the release of IL-5 by activated lymphocytes and increases the expression of adhesion molecules by endothelial cells further promoting the recruitment, activation, and proliferation of eosinophils (112, 119–122). The resulting eosinophilia observed in EoE is a common feature of most members of the atopic march. In EoE, as well as in AD, BA and CRSwNP, the dysregulated release of cytotoxic proteins by eosinophils ultimately causes tissue damage and barrier dysfunction thus promoting epithelial remodeling (92, 119, 120, 122).

Conclusively, an ALOX15 + macrophage subset driving M2 polarization was recently described also in EoE, (123) similarly to CRSwNP, in which IL-4/IL-13/ALOX15 M2 macrophages play a pivotal role (124), thus supporting the aforementioned role of macrophages in T2 inflammatory diseases.

The previously mentioned molecular mechanisms endorse the rationale for the development of biologic drugs with targets shared by multiple diseases.

### 3.3 Non-shared immunologic pathways

Despite common pathogenetic mechanisms, some biologic agents have demonstrated their efficacy in other T2 diseases but not in EoE (Table 2) (8). This supports the hypothesis that while hitting some T2-related pathways may be sufficient to control the inflammatory process developed in specific atopic diseases, the block of these pathways may be irrelevant to control EoE.

**TABLE 2:** Biological drugs approved for other T2 diseases also investigated in EoE (8).

Drug	Target	T2-diseases with approval	Completed/Ongoing trials in EoE	Results in EoE	
Omalizumab	IgE	BA, CRSwNP (FDA, EMA)^1^	Completed	Histological and clinical improvement only in 33% of patients	(137)
Mepolizumab	IL-5	BA, CRSwNP (FDA, EMA)^1^	Completed	Significant reduction in esophageal eosinophilia but never remission (defined as <5 eos/hpf). No symptomatic improvement	(131)
Dupilumab	IL-4/IL-13	BA, CRSwNP, AD, EoE (FDA, EMA)	Completed in pts ≥12 y.o. Completed in children 1-11 y.o.	Improved histologic and clinical outcomes; DSQ improved only in weekly administration. Significant reduction in eosinophilic count ≤6 eos/hpf; endoscopic and clinical improvement	(149, 150)
Reslizumab	IL-5	BA (FDA, EMA)	Completed	Significant reduction in esophageal eosinophilia. No clinical improvement	(129)
Tezepelumab	TSLP	BA (FDA, EMA)	Ongoing (NCT05583227)	n.a.	
Benralizumab	IL-5Rα	BA (FDA, EMA)	Completed	Significant reduction in esophageal eosinophilia. No clinical improvement	(130)

BA, bronchial asthma; CRSwNP, chronic rhinitis with nasal polyps; FDA, food and drug administration; EMA, European medical agency; AD, atopic dermatitis; EoE, eosinophilic esophagitis; DSQ, dysphagia symptom questionnaire; pts, patients; TSLP, thymic stromal lymphopoietin; IL-5Rα, Interleukin 5 Receptor α.

Although eosinophilic inflammation has a crucial role in the pathophysiology of EoE, experimental data support the presence of other eosinophils-independent mechanisms. Several studies outlined that IL-13 overexpression alone is able to promote the expression of global EoE-related transcriptome (103, 116, 125, 126), thus IL-13 stimulation could be sufficient to promote disease development. This concept is supported by data from eosinophil lineage-deficient IL-13 transgenic mice which develop an eosinophils-independent EoE-like phenotype with esophageal remodeling induced by IL-13 overexpression (116). Therefore, unlike other atopic diseases, blocking the eosinophils-driven inflammation alone may not be sufficient to control the inflammatory process operating in EoE. This is further confirmed by the results obtained with anti-IL-5 antibodies, already approved in other T2 diseases (127). Indeed, in EoE, anti-IL-5 antibodies have so far shown to be effective in reducing eosinophilic count but not in symptom resolution (128–131).

IgE hyperproduction by activated B cells, together with basophils and mast cells degranulation, play a pivotal role in T2 diseases (132–134). In EoE, despite mast cells have an active role, (125, 135, 136) it seems to be unrelated to IgE-mediated degranulation, as supported by the evidence that anti-IgE antibody showed to be ineffective in EoE patients (137). Accordingly, EoE is now considered a non-IgE-mediated type 2 inflammatory disease, departing from IgE-mediated FA and other atopic conditions (138).

In patients affected by multiple atopic disease in association with EoE, these evidence support a therapeutic approach based on the selection of a molecular target in a shared area of the inflammatory cascade located upstream in the T2 immunologic pathway.

## 4 Dupilumab and other therapeutic frontiers in the “atopic march”

### 4.1 Dupilumab: how does it work

Dupilumab is a fully humanized IgG4 antibody targeting the α-chain of the IL4R. There are two different types of IL-4R: the type I is composed by IL-4Rα and the common γ chain, while IL-4Rα and the α-1 chain of IL-13 (IL-13Rα1) form the type II receptor. Consequently, type I receptor can only bind IL-4, while type II receptor is able to bind both IL-4 and IL-13 (110, 139). Thus, dupilumab, by blocking IL-4Rα is able to interrupt the T2 inflammatory process (108).

### 4.2 Dupilumab in atopic diseases

The Food and Drug Administration (FDA) first approved dupilumab (Dupixent) in 2017 for AD, in patients 18 years and older (140, 141). In subsequent years, dupilumab has been also approved for AD in adolescents 12–18 years old, in children 6–11 years old and finally in children 6 months to 5 years old (142–144). First dupilumab approval for BA in children 12 years and older and in adults is dated 2018, (145, 146) and in 2021 FDA expanded its approval also for BA in children 6–11 years old (147). In 2019 dupilumab received approval in CRSwNP patients older than 18 years (148). Finally, in 2022, the FDA approved dupilumab for EoE in patients older than 12 years (149). Most recently, in January 2024, the FDA recognized dupilumab as the first and only treatment for children aged 1 year and older affected by EoE (150, 151).

The European Medicines Agency (EMA) currently approves dupilumab use for moderate to severe AD in patients aged 12 years and older non-responsive to conventional treatment and for AD children of 6–11 years of age with severe disease. Dupilumab is also approved by EMA for severe refractory asthma in patients aged at least 6 years, for adults patients affected by CRSwNP, and for EoE in adults and children over 1 year of age unresponsive to, or not able to take conventional first-line therapies (152).

Real word data on the efficacy of dupilumab in the non-EoE members of the “atopic march” are currently available. An interim analysis of the real-world PROSE registry from the US and Canada, (153) counting a total of 764 adolescents and adults patients affected by moderate-to-severe AD treated with dupilumab, showed a persistence on therapy in 83% of patients after 2 years of follow up. Improvements in all clinician and patient reported outcome were observed within the first 3 months of therapy and maintained throughout 24 months.

The recently published retrospective study US ADVANTAGE included 2400 patients older than 12 years affected by moderate-to-severe asthma treated with dupilumab (154). After 1 year of treatment, the risk of asthma exacerbation was reduced by 44% and the number of systemic corticosteroid prescriptions by 48%.

Real-world data on CRSwNP come from an observational Phase IV real-life study (DUPIREAL), which assessed the effectiveness and safety of dupilumab in 648 patients with severe uncontrolled CRSwNP over the first year of treatment (155). The authors reported a significant improvement in nasal polyps score (NPS), assessed as primary outcome; but secondary outcomes such as symptoms and olfactory function were also improved. Moreover, disease control based on EPOS 2020 criteria was observed in 96.9% of patients at 12 months with a moderate to excellent response (156).

These real-world data confirm the efficacy and safety of dupilumab in AD, BA and CRSwNP. The most frequent adverse events (AEs) were conjunctivitis, rhinopharyngitis, arthralgia and upper airways infections that in most cases do not led to treatment discontinuation (153, 155, 157). However, despite the majority of included patients presented at least another T2 atopic comorbidity, all of these studies have been designed to focus on the efficacy of dupilumab on the main indication, and no sub-analysis evaluating its efficacy on the comorbid atopic diseases was made.

### 4.3 Dupilumab in EoE

In the phase III randomized controlled trial (RCT) by Dellon et al. (149) (LIBERTY EoE TREET) on EoE patients ≥12 years old, 81 patients in part A (dupilumab 300 mg s.c. weekly vs placebo) and 240 patients in part B (dupilumab 300 mg s.c. weekly or every other week (eow) vs placebo) were included. Both part A and B were designed for 24 weeks duration, whereas in part C treatment was extended up to 52 weeks as maintenance. Weekly dupilumab was effective to induce histological remission (defined as esophageal eosinophilic count of <6 eos/hpf) in 60% and in 59% of patients in part A and part B, respectively. Similar results were achieved with the eow administration. However, the clinical outcome, measured by the absolute change in the Dysphagia Symptom Questionnaire (DSQ) score from baseline, was achieved only with the weekly administration of dupilumab. The extension study confirmed weekly dupilumab to be effective in maintaining both clinical and histological remission at week 52 (149, 158). It is worth noting that more than two thirds of enrolled patients had a difficult-to-treat disease, considering that up to 74% of cases was refractory to multiple previous therapies and that almost one third of patients had a fibro-stenotic phenotype, presenting a mean of two previous esophageal endoscopic dilations (149, 158).

Given the more recent approval of dupilumab in EoE, real-world data on its effectiveness in clinical practice are scarce compared with other T2 diseases. A recent retrospective cohort study by Lee and Dellon (159) included 46 adults patients treated with dupilumab for severe, refractory, fibro-stenotic EoE. After a median of 6 months of dupilumab therapy, the authors reported a significant improvement in endoscopy, histology, symptoms, and hrQoL. In particular, after dupilumab histological remission (defined as <15 eos/hpf) was achieved in up to 80% of cases, EREFS score decreased significantly from baseline and esophageal stricture diameter significantly increased.

The phase III RCT trial EoE KIDS recently reported data on efficacy and safety of dupilumab in 102 EoE patients 1–11 years of age, failure to PPI therapy (150). Patients were randomized into high and low exposure to dupilumab groups, reproducing the weekly and the eow dosage of the LIBERTY EoE TREET and to a placebo group. The primary end point was histologic remission (defined as peak eosinophilic count ≤6 eos/hpf) at week 16. After 16 weeks, all patients could enter 36 weeks extended active treatment period and a 108 weeks open-label extension period. Compared to placebo, the primary endpoint was met by significantly more patients in both dupilumab exposure groups (68% high exposure, 58% low exposure vs 3% placebo). However, statistically significant symptoms’ improvement, changes in the EREFS score and in the EoEHSS were observed and maintained through week 52 only in dupilumab high-exposure group.

The safety profile of dupilumab in EoE founded to be consistent with other atopic disease, both in adults and children. Injection site reactions, rhinopharyngitis, headache and nausea were the more frequent reported AEs and rarely led to therapy discontinuation (150, 158).

## 5 Dupilumab as an umbrella treatment in the “atopic march”: real world evidence

The aforementioned pivotal studies and real-world experiences clearly showed the efficacy of dupilumab in multiple atopic diseases including EoE. Notably, in these studies many enrolled patients were affected by multiple T2 diseases. In the real-word PROSE study (153) 58.6% of patients at baseline reported another T2 inflammatory comorbid disease in addition to AD. In the US ADVANTAGE retrospective study (154). 93.9% of patients affected by asthma had at least one additional ongoing atopic medical condition. In the phase IV real-world study DUPIREAL (155) on CRSwNP, 56.5% of patients reported concomitant BA. In studies exploring the efficacy of dupilumab in EoE, 84% of the study population had concomitant mild-to-moderate type 2 inflammatory diseases, including AR (59.3%), FA (44.4%), BA (30.9%), and AD (18.5%) (149, 158). Eventually, 100% of children enrolled in the phase III trial EoE KIDS reported atopic comorbidities (150). However, none of these studies evaluated the efficacy across indications in the same patients. A retrospective observational study by Napolitano et al. (160) evaluated the efficacy of dupilumab in the management of concomitant AD and CRSwNP. The authors concluded that dupilumab, besides confirming to be effective in improving significantly both AD and CRSwNP outcomes, increased patients’ adherence and showed benefits in term of health-care related costs. Similarly, in a recent prospective, multicenter, observational, real-world study by Caminati et al. (161), the effectiveness and safety of dupilumab in BA patients with or without CRSwNP were assessed throughout 12 months of follow-up. Results from the subgroup of patients with concomitant BA and CRSwNP showed an improvement in both BA and CRSwNP-related outcomes, supporting the rationale for using dupilumab for the management of multiple concomitant atopic diseases in the same patient.

To the best of our knowledge, there are currently no RCT or large prospective studies investigating the efficacy of dupilumab on multiple co-existing atopic disease in association with EoE. Evidence of efficacy of dupilumab in these patients only comes from small studies and case reports.

The first case report about the use of dupilumab in treating multiple atopic diseases only appeared in 2020. The authors described a 9-years-old boy suffering from multiple treatment-refractory AD with concomitant severe BA and EoE refractory to conventional therapies, who was enrolled in a phase III RCT of dupilumab designed for AD children 6 to 12-years-old (162). After the administration of dupilumab 200 mg eow, in addition to a marked AD improvement the boy was also able to reduce his antihistamine medications and the use of inhaled CS to as-needed basis. He also reported less dysphagia, in accordance with repeat esophageal biopsy specimens that showed 0-5 eos/hpf in all studied segments.

Chawla et al. (163) reported a case of a 42-years-old man affected by EoE in conjunction with chronic urticaria and allergic rhinitis. Despite previous treatment with PPIs, 6-FED and STCs, he exhibited ongoing symptomatic and histologic activity (>50 eos/hpf) and ultimately presented with food impaction and deep esophageal ulcerations. His chronic urticaria was controlled by Omalizumab, while his allergic rhinitis, similarly with EoE, had suboptimal symptomatic control despite multiple therapies. Taking into consideration all his polypharmacy, Omalizumab was stopped, and dupilumab was started after multidisciplinary discussion. After 12 weeks of dupilumab treatment, he reported a symptomatic improvement in all his comorbidities and obtained EoE histologic remission (<10 eos/hpf) on repeat EGD.

Another case-report describing a 14-years-old boy having severe AD-Hyper IgE Eosinophilic Syndrome (AD-HIES) with frequent flares not responding to conventional therapy, was presented by Dixit et al. (164) The patient had concomitant symptomatic EoE treated unsuccessfully with STCs and PPIs, as the follow-up repeat endoscopy revealed an eosinophilic infiltrate of 135 eos/hpf. Dupilumab loading dose, followed by maintenance with 300 mg every 2 weeks was prescribed for the poorly controlled AD-related eczema. After 8 weeks of treatment, AD index fell dramatically, and dysphagia almost completely resolved. Repeat endoscopy at week 6 revealed 6 eos/hpf.

Finally, one case report on a patient with multiple T2 comorbidities (i.e., BA, EoE, AD, CRSwNP), in whom asthma was the primary indication for dupilumab prescription, was published in 2023 (165). After 12 months of treatment, an improvement in the clinical parameters of all the concomitant diseases was observed. Moreover, the authors stressed on the efficacy of dupilumab in significantly increasing the objective measurement of hrQoL across all four comorbidities.

In a recently published real-world case series, 7 patients receiving subcutaneous dupilumab every 2 weeks prescribed for asthma and/or AD as primary indications, had concomitant EoE, mostly with a severe phenotype (166). The median age was 15.8 years (IQR 9.3–19.5 years) and all patients had already failed conventional therapy Indeed, before dupilumab initiation, the median peak esophageal eosinophil count at biopsies was 50 eos/hpf (IQR 48-95 eos/hpf). After dupilumab, all patients reported of less frequent asthmatic exacerbations and were able to decrease albuterol and inhaled corticosteroid use. Moreover, they complained of fewer AD exacerbations. Concerning EoE, all patients obtained histological remission with a median peak esophageal eosinophil count of 2 eos/hpf (IQR 0-5 eos/hpf) at the endoscopic follow-up performed after a median of 5.3 months. Of note, all patients had waned off STCs and had successfully re-introduced the previously excluded food elements in their diet.

Finally, Sauer et al., (167) reported 2 cases where dupilumab was considered to be an ideal choice to treat concomitant T2 atopic conditions. The first case described a 14-year-old girl with a history of severe AD, persistently active BA and seasonal AR. After two episodes of food impaction, an EGD was performed, revealing the presence of >50 eos/hpf at biopsies, The second case described a 39-year-old man with a history of AR and fibro-stenotic EoE resistant to multiple conventional therapies, as showed by a persistent eosinophilic count >50 eos/hpf on biopsy specimens despite PPI, STCs and FED. In both cases dupilumab was considered the first shared therapeutic choice, obtaining clinical, histologic (<6 eos/hpf) and endoscopic remission (evaluated at 3–5 months after dupilumab induction) in each case.

In a retrospective chart review by Spergel et al. (168), all the 45 included pediatric patients in which dupilumab had been prescribed for BA, AD or CRSwNP as primary indication, reported a concomitant diagnosis of EoE. Despite the dupilumab eow administration, histologic improvement (defined as peak eosinophilic count ≤6 eos/hpf) was reported in 22 out of 26 patients in which repeat histology was available, while clinical improvement was reported in 28 patients, of which 24/28 had a complete resolution of symptoms after dupilumab initiation. Of note, 29 patients were able to reduce other EoE medications (PPIs, STCs, FED).

In a recent small retrospective study by Tomàs-Peréz et al. (169) 15 patients older than 18 years received dupilumab 300 mg eow for BA or AD, (9 and 6 patients, respectively) all had concomitant diagnosis of EoE, and 8 patients had concomitant CRSwNP (BA: 4; AD: 4). All patients who received dupilumab for more than 6 months, showed improvement in all concomitant atopic diseases.

## 6 Discussion and future perspectives

EoE is a chronic T2 inflammatory disease that only recently has been recognized as a member of the “atopic march” (3). The grouping of these diseases relies on shared similarities at the epidemiological, genetic, environmental and pathogenetic level. Indeed, multiple T2 diseases frequently co-exist in the same patient. Considering the natural history of these diseases, characterized by a chronic relapsing-remitting course, an efficient long-term maintenance therapy is required. Nevertheless, the management of T2 inflammatory diseases is still challenging, since many conventional therapies fail to induce and/or maintain remission or to prevent disease exacerbations and complications in the long-term. Moreover, these diseases are often treated without a shared multidisciplinary approach, often causing polypharmacy and steroids chronic use (4, 5). For all these reasons, various biological drugs targeting shared T2-related pathways (including IL-5, IL-4/IL-13, IgE, TSLP) have been developed (8). These drugs, namely omalizumab, mepolizumab, reslizumab, benralizumab, dupilumab and tezepelumab, have been firstly approved for the treatment of other atopic diseases and subsequently evaluated also in EoE. However, to date, dupilumab is the only biologic agent to have shown efficacy in inducing clinical and histological remission in EoE. Accordingly, it is the only biologic drug that has received approval by both FDA and EMA for the treatment of EoE, even though with a weekly administration therapeutic scheme compared with eow administration approved in other atopic diseases (151, 152).

**FIGURE 2:**
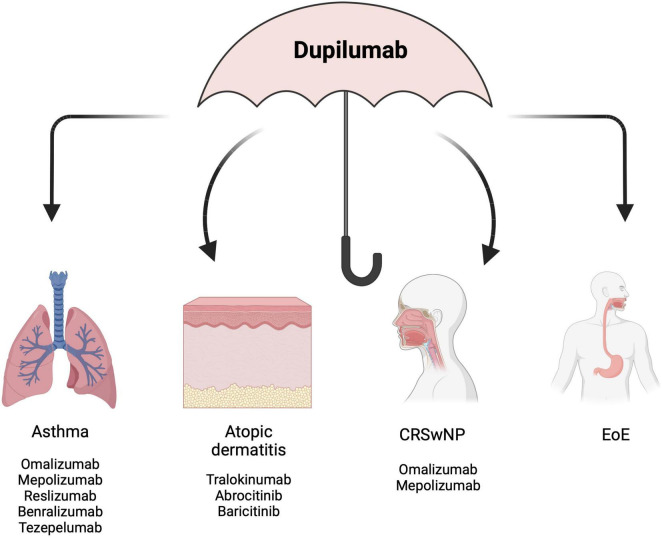
Dupilumab as an “umbrella” to treat multiple co-existing diseases. EoE, eosinophilic esophagitis; CRSwNP, chronic rhinitis with nasal polyps. Created with BioRender.com.

Considering the effectiveness of dupilumab in the management of each of the T2 allergic diseases belonging to the “atopic march”, it is highly reasonable to hypothesize that dupilumab might be used as a single therapy across multiple indications in the same patient (Figure 2). In support of this rationale, Aceves et al. and the most recent Italian consensus EoETALY, highlighted the possibility of dupilumab use as a first-line approach when EoE co-exists with other T2-related comorbidities (58, 170). Snyder and Dellon (171) suggested that dupilumab should be placed in treatment algorithms not only in EoE patients who are PPI or STC non-responders, but also as first-line therapy in patients having multiple atopic comorbidities. Moreover, the early recognition of EoE “red flags” by immunologists and dermatologists and the early referral to the gastroenterologist may reduce diagnostic delay and long-term complications and may offer the opportunity to use dupilumab earlier in the course of the disease, in a “top-down approach” that already has shown benefits in other chronic conditions (172). Considering dupilumab as the most appropriate treatment in those patients affected by EoE in addition to other T2 atopic diseases, a shared-decisional approach may gauge the scheme of treatment based on the most clinically relevant disease. However, studies designed to assess the effectiveness of dupilumab for the treatment of multiple T2 comorbidities are currently lacking, partly due to the absence of standardized framework for evaluating dupilumab efficacy across multiple T2 diseases. Indeed, despite the high percentage of concomitant atopic diseases reported in the population of the RCTs and real-world studies (140–150, 153–155, 157, 158, 162–166, 173), the efficacy of dupilumab across all comorbidities was not evaluated in *post hoc* analysis or sub-analysis. However, data originating from the retrospective study by Caminati et al. (161) in which dupilumab was prescribed primary for BA, in patients with or without concomitant CRSwNP, outlined the effectiveness of dupilumab in improving both BA and CRSwNP-related outcomes supporting the efficacy of dupilumab across multiple indications in the same patients.

Evidence on the beneficial effect of a multidisciplinary approach in the management of patients affected by multiple immune-mediated diseases sharing similar immunologic pathways comes from other fields. Lucchetti et al. (174) demonstrated that a multidisciplinary management of patients with inflammatory bowel disease-associated spondyloarthritis, significantly improved both articular and intestinal disease activities in addition to patients’ hrQoL. Indeed, gastroenterologists, rheumatologists and dermatologists often discuss therapeutic options together in a multidisciplinary setting, in order to choose one biologic agent able to cover multiple co-existing chronic inflammatory diseases avoiding polypharmacy. Although this concept has been widely explored in other clinical scenarios, similar solid data in the “atopic march” diseases are still scarce. Considering the different number of therapeutic options available for the T2 diseases, a case-by-case multidisciplinary evaluation could be reasonable, in order to target multiple diseases as an “umbrella” therapeutic strategy, hence avoiding the prescription of multiple drugs, reducing useless excessive sanitary costs, improving both patients’ hrQoL and adherence to therapy. Indeed, dupilumab showed a higher cost-effectiveness if compared to other biologics and conventional therapy in BA. Moreover, recent data by Ong et al. (175) demonstrated that dupilumab was cost-effective compared to JAK inhibitors in AD (176–179). Although when considering each disease separately, dupilumab has not been found to be the best choice in terms of cost-utility as first-line therapy, it seems reasonable to hypothesize that the use of dupilumab as first line treatment of multiple concomitant T2 comorbidities may be cost-effective compared to polypharmacy, especially when multiple biological drugs are prescribed in the same patient. Moreover, real-world data showed higher adherence rates to Dupilumab than to conventional therapy (180–182). Although the safety profile of dupilumab in each disease is well known, (150, 153, 155, 157, 158) data concerning safety of dupilumab as “cross-therapy” in patients with concomitant T2 comorbidities are lacking. Nevertheless, it is reasonable to hypothesize that a single therapy could be associated with less AEs compared to polypharmacy.

This concept is even stronger in the pediatric population. The earlier onset of EoE in children already diagnosed with other atopic diseases remarks the link between EoE and atopic march. Of note, combination therapy is frequently needed to achieve remission in early-onset EoE, hence contributing to higher risk of polypharmacy (183, 184). Furthermore, although the therapeutic efficacy of dupilumab is well documented, its role in interrupting the “atopic march” and in preventing its evolution remains unclear. Recently, Lin et al. (185) investigated the contribution of dupilumab in the atopic march progression. More than 4 thousand pediatric AD patients were categorized in two cohorts: one cohort, receiving early dupilumab prescription (DUPI-cohort) and one cohort who received conventional immunomodulators (CONV-cohort). The 3-year cumulative incidence of atopic march progression was significantly lower in the DUPI-cohort respect to the CONV-cohort, as well as the risk of developing BA and AR. However, due to the retrospective design of the study, the direct causality cannot be established, and prospective data are urgently needed to explore the theoretical possibility of modifying the natural history of T2 diseases.

Finally, evidence on biomarkers and standardized tools predictive of treatment response in multidisciplinary care is lacking (183). Future research should focus on these unmet needs, thus moving toward a precision medicine and a more treat-to target strategy.

In conclusion, the blockage of IL4/IL13 signaling operated by Dupilumab may represent an effective strategy that could be used as first line therapy in patients affected by EoE in association with other T2 diseases.
