# Supplementary material for: Eosinophilic esophagitis in the “atopic march”: dupilumab as an “umbrella” strategy for multiple coexisting atopic diseases
Source: Front Med (Lausanne). 2025 Jan 21;11:1513417. doi: 10.3389/fmed.2024.1513417 (PMC11790572; doi:10.3389/fmed.2024.1513417)
Supplement: Supplementary file 1 [file Table_1.docx]

Supplementary Material

# Supplementary Data

Search strategies:

*A search was conducted across Medline database, using a permutation of key words as “dupilumab”, “eosinophilic esophagitis”, “EoE”, “atopic dermatitis”, “AD”, “bronchial asthma”, “BA”, “allergic rhinitis”, “AR”, “chronic rhinosinusitis with nasal polyps”, “CRSwNP”, “atopic march”, “atopic diseases”, “alarmins”, “TSLP”, “IL-4”, “IL-13”, “eotaxin-3”, “IL-5”, “eosinophils”, “epithelial barrier”, “mepolizumab”, “omalizumab”, “benralizumab”, “reslizumab”, “tralokinumab”, “multidisciplinary care”, “safety”, “adherence”. Then, a search was conducted across references of selected papers*
